# Supplementary material for: Obesity drives depot-specific vascular remodeling in male white adipose tissue
Source: Nat Commun. 2025 Jun 25;16:5392. doi: 10.1038/s41467-025-60910-2 (PMC12198381; doi:10.1038/s41467-025-60910-2)
Supplement: Supplementary file 1 — Supplementary information [file 41467_2025_60910_MOESM1_ESM.pdf]

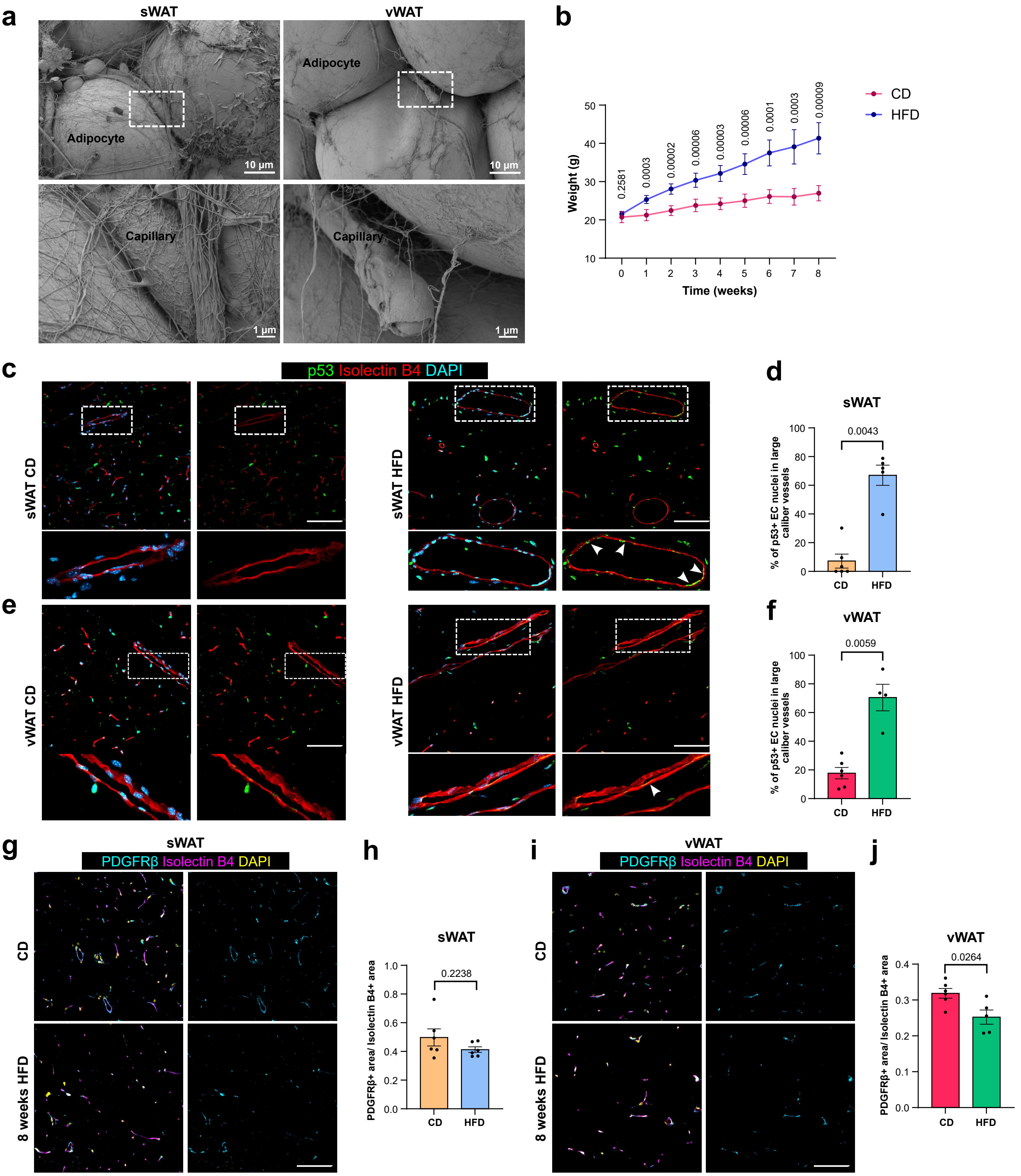

Supplementary Figure 1: Characterization of blood vessels in sWAT and vWAT.

a, Representative scanning electron microscopy images of subcutaneous (sWAT) and visceral (vWAT) white adipose tissue. **b,** Weight curve of mice kept on control (CD) or high fat diet (HFD) for 8 weeks (n=8 mice in each group). **c,** Representative confocal images of p53 (green), isolectin B4 (red) and DAPI (blue) staining on sWAT sections and **d,** quantification of p53+ endothelial cell (EC) nuclei in large vessels (CD n=6, HFD n=5). **e,** Representative confocal images of p53 (green), isolectin B4 (red) and DAPI (blue) staining on vWAT sections and **f,** quantification of p53+ EC nuclei in large vessels (CD n=6 mice, HFD n=4 mice). **g,** Representative confocal images of PDGFRβ (cyan), isolectin B4 (magenta) and DAPI (yellow) staining on sWAT sections from lean (CD) and obese (HFD) mice. **h,** Quantification of PDGFRβ area around blood vessels normalized to isolectin B4+ area in sWAT (n=6 mice in each group). **i,** Representative confocal images of PDGFRβ (cyan), isolectin B4 (magenta) and DAPI (yellow) staining on vWAT sections from lean (CD) and obese (HFD) mice. **j,** Quantification of PDGFRβ area around blood vessels normalized to isolectin B4+ area in vWAT (lean n=6, obese n=5 mice). Scale bars 100 µm. Data represents ± SEM, two-sided Mann-Whitney test (d), two-sided Welch's t-test (b, f, h, j). Source data are provided as a Source Data file.

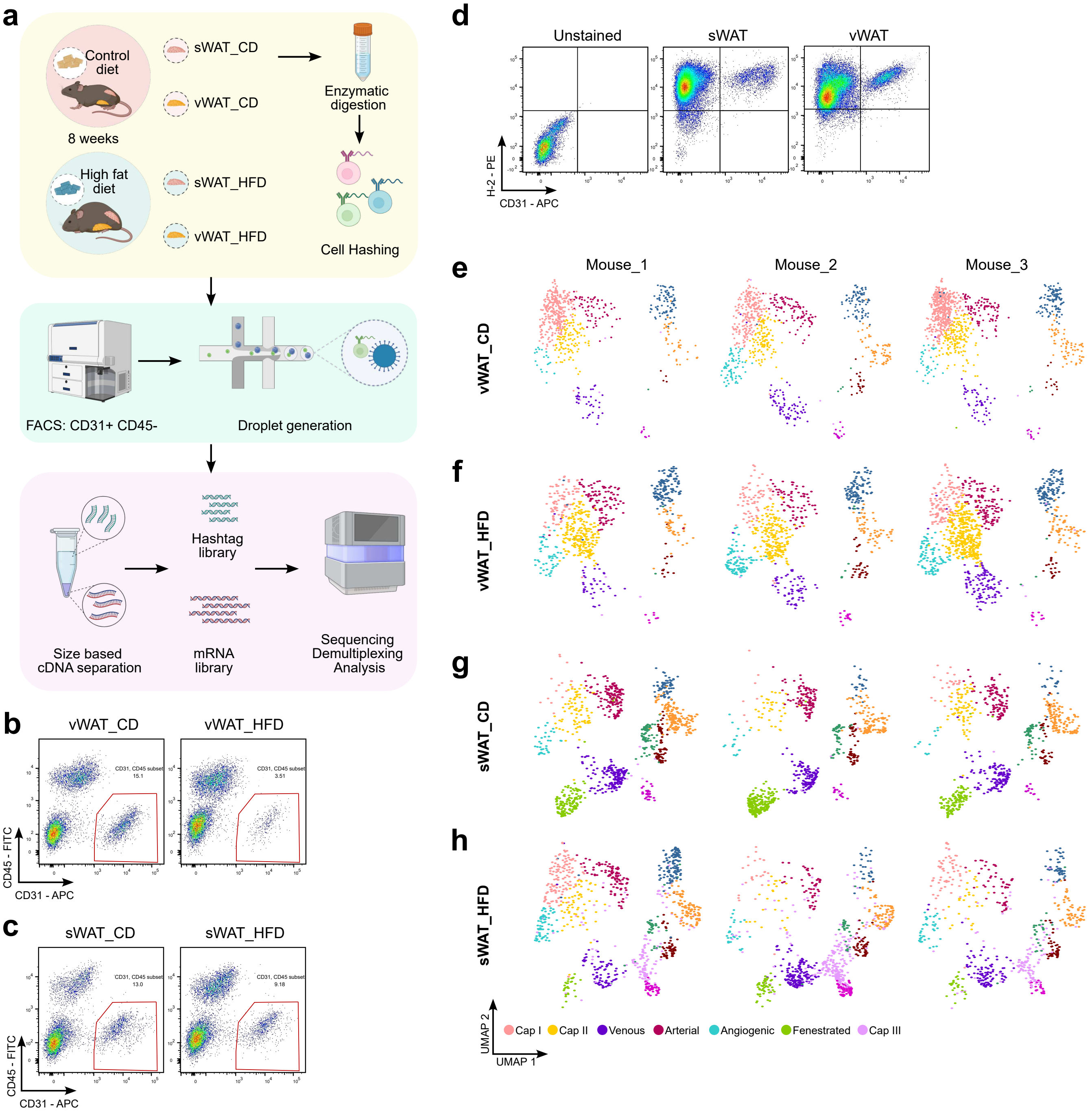

Supplementary Figure 2: EC isolation strategy and demultiplexed cell hashing.

a, Schematic representation of experimental design for single-cell transcriptomics. Schematics created in BioRender. Hasan, S. (2025) https://BioRender.com/uwlgadm. **b,** Representative FACS plots showing sorting strategy for CD31+ CD45- endothelial cells (ECs) from visceral white adipose tissue (vWAT) or **c,** subcutaneous WAT (sWAT). **d,** FACS plot depicting labeling of CD31+ cells with MHC Class I antibody (H2-PE) used for cell hashing. **e-h** Demultiplexed data after quality control and assigning respective hashtags to separate the biological replicates in different depots in lean (control diet, CD) and obese (high fat diet, HFD) mice.

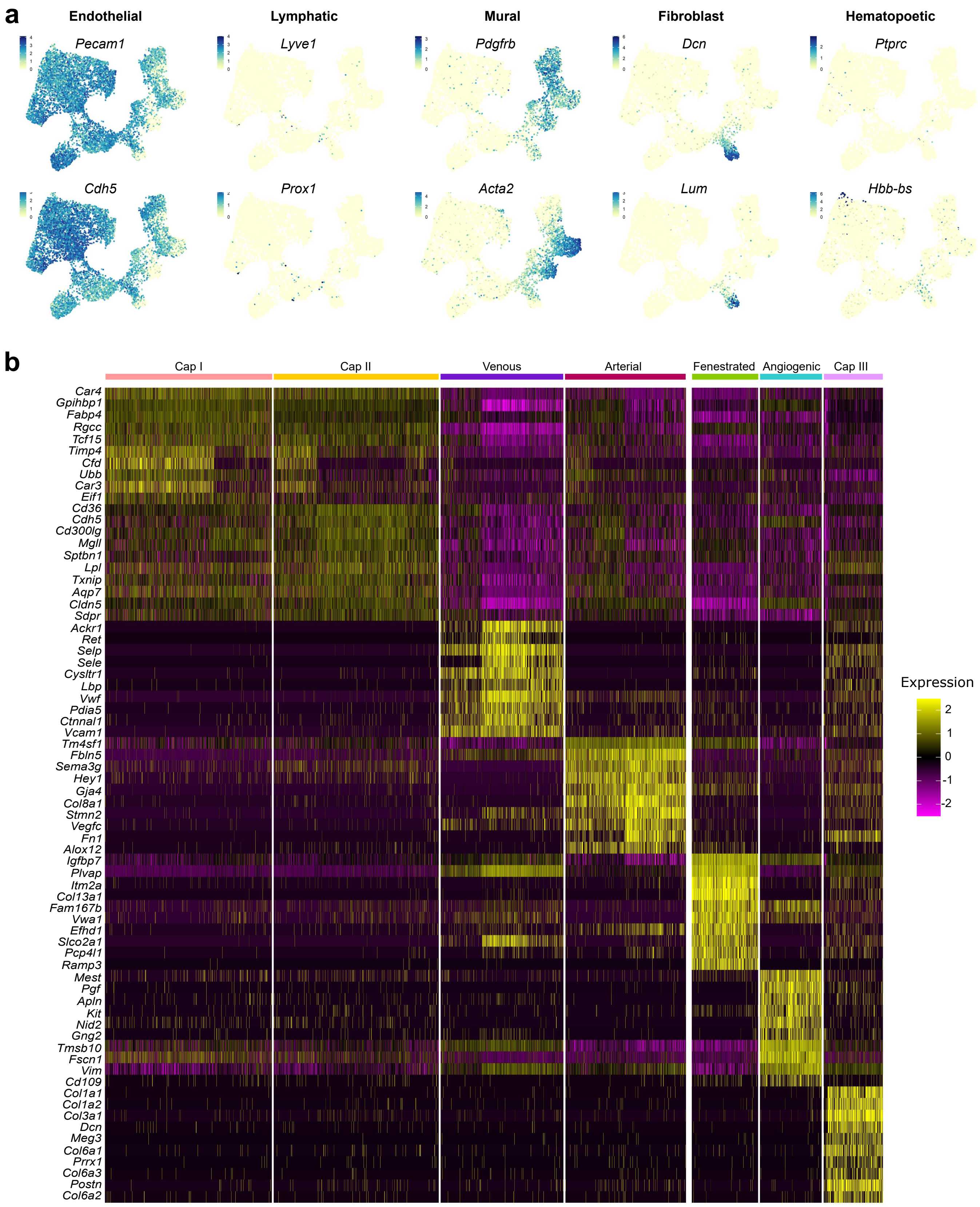

Supplementary Figure 3: Marker gene expression in different clusters.

a, UMAP plots showing expression of marker genes selective for endothelial, lymphatic, mural, fibroblast and hematopoietic cells. **b,** Heat map showing top 10 marker gene expression across different clusters.

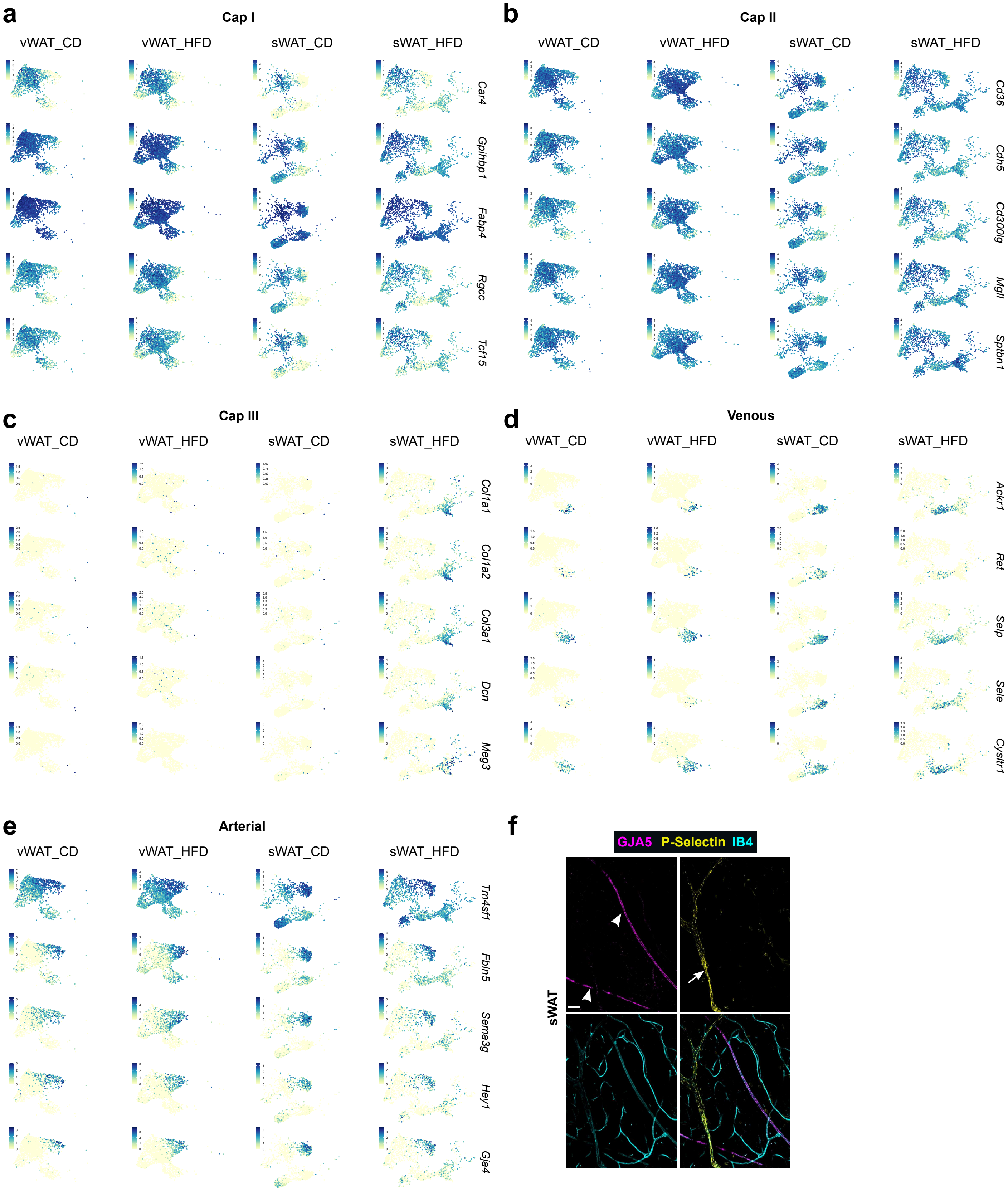

Supplementary Figure 4: Marker gene expression in different cluster under different conditions

UMAPs depicting representative marker expression in **a,** Cap I, **b,** Cap II, **c,** Cap III, **d,** venous and **e,** arterial endothelial subtypes in subcutaneous (sWAT) and visceral white adipose tissue (vWAT) in lean (control diet, CD) and obese (high fat diet, HFD) mice. **f,** Representative orthogonal projections of sWAT stained for GJA5 (magenta, arterial marker, arrowhead), P-selectin (yellow, venous marker, arrow) and isolectin B4 (cyan, capillary). Scale bar 50 µm.

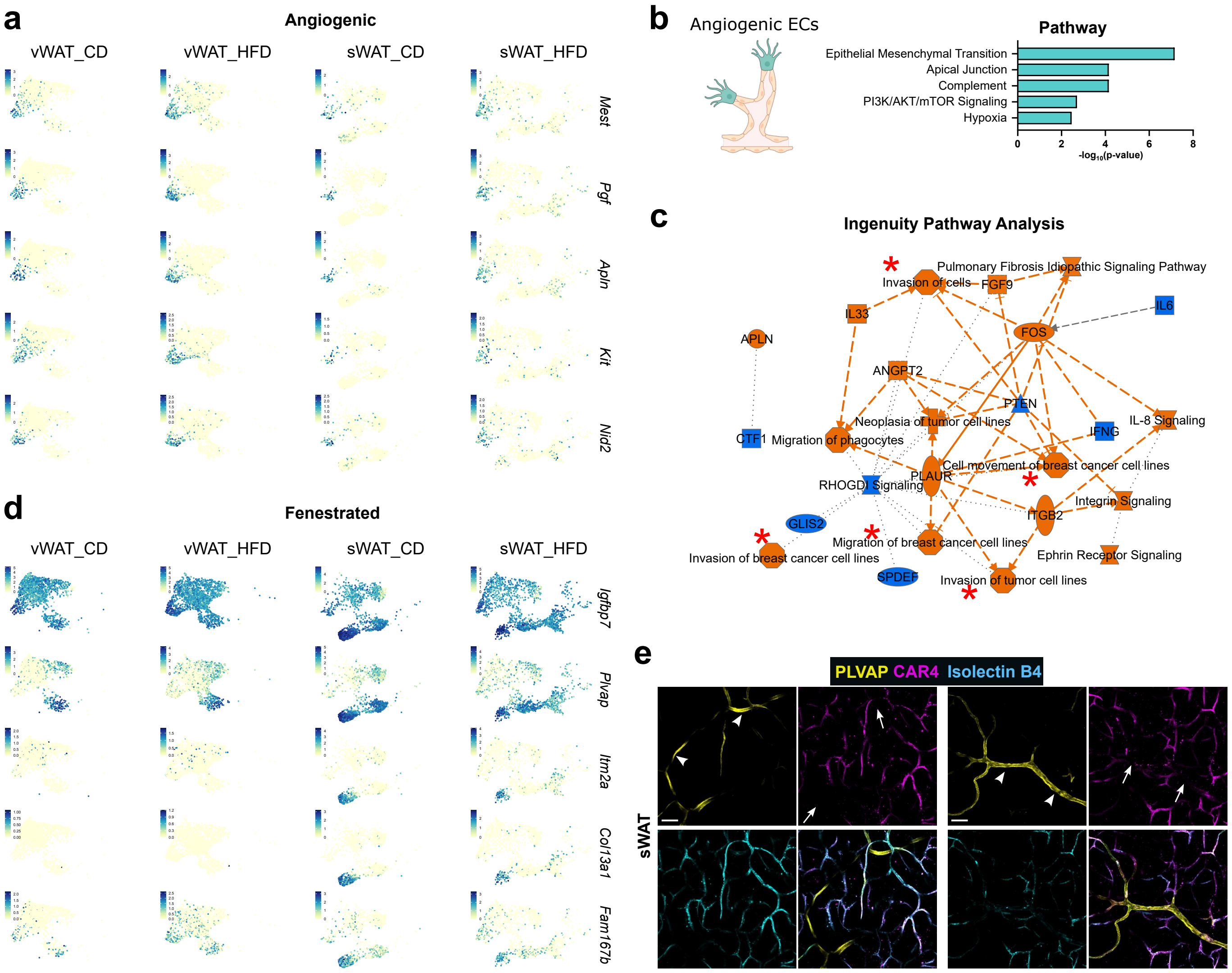

Supplementary Figure 5: Angiogenic and fenestrated endothelial cells in WAT.

a, UMAP plots showing enriched marker gene expression in angiogenic endothelial cells (ECs) from subcutaneous (sWAT) and visceral white adipose tissue (vWAT) under lean (control diet, CD) and obese (high fat diet, HFD) conditions. **b,** Upregulated pathways in angiogenic ECs. Schematic created in BioRender. Hasan, S. (2025) https://BioRender.com/5n7dalg. **c,** Ingenuity pathway analysis (IPA) on upregulated marker genes in this cluster. Asterisk denotes nodes highlighting invasive and migratory behavior. Orange and blue graphics represent predicted activation and inhibition respectively. Straight lines and dotted lines represent direct or indirect interaction respectively. **d,** UMAP plots showing enriched marker gene expression in fenestrated ECs under different conditions. **e,** Representative orthogonal projections from whole mount staining for PLVAP (yellow, arrowheads), CAR4 (magenta, arrows), and isolectin B4 (cyan) in sWAT. Scale bar 50 µm. Source data are provided as a Source Data file.

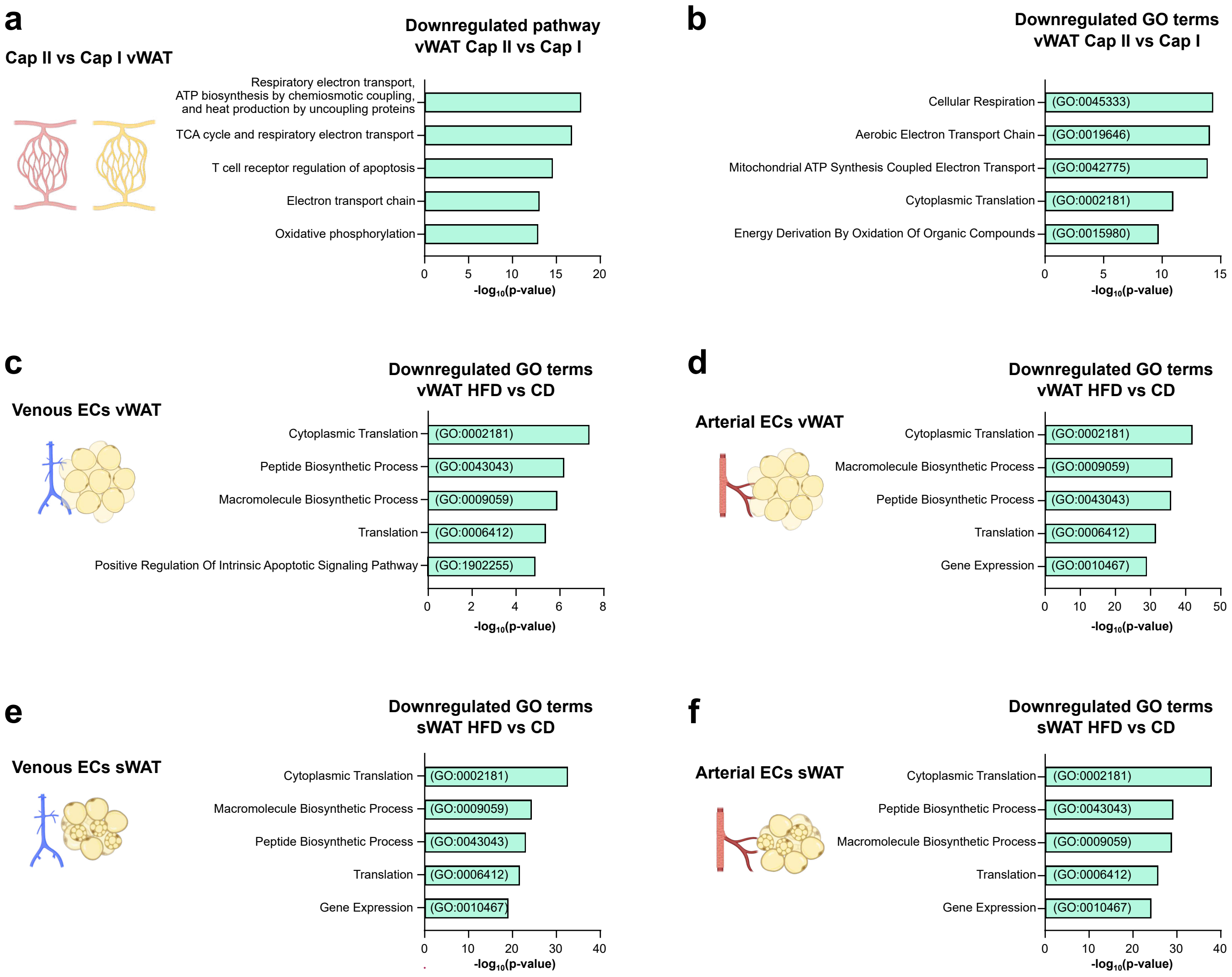

Supplementary Figure 6: Obesity driven downregulated pathways and GO terms.

a, Bioplanet annotated downregulated pathways in Cap II endothelial cells (ECs) compared to Cap I ECs in visceral white adipose tissue (vWAT). **b,** Gene ontology (GO) terms downregulated in Cap II ECs compared to Cap I ECs in vWAT. **c,** Downregulated GO terms in obese (high fat diet, HFD) vWAT venous ECs compared to lean (control diet, CD). **d,** Downregulated GO terms in obese vWAT arterial ECs. **e,** Downregulated GO terms in obese subcutaneous WAT (sWAT) venous ECs. **f,** Downregulated GO terms in obese sWAT arterial ECs. All schematics created in BioRender. Hasan, S. (2025) https://BioRender.com/ossmape. Source data are provided as a Source Data file.

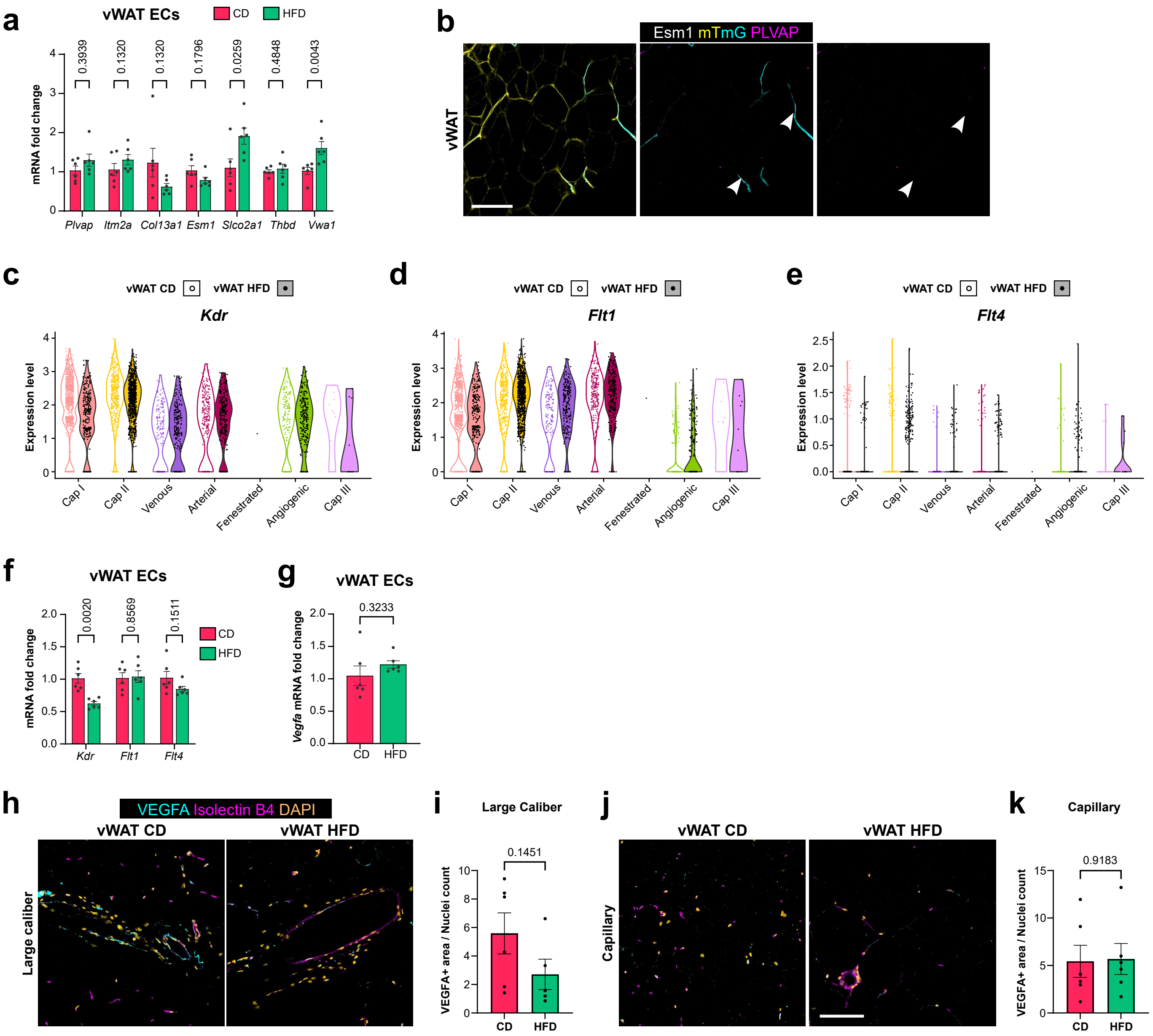

Supplementary Figure 7: Characterization of fenestrated endothelium.

a, Quantitative real-time PCR detection of marker genes of fenestration in visceral white adipsoe tisssue (vWAT). Endothelial cells (ECs) isolated from lean (control diet, CD n=6 mice) and obese (high fat diet, HFD n=6 mice) animals. **b,** Representative orthogonal projections from whole mount staining for PLVAP (magenta) performed on vWAT from Esm1:Cre-ERT2 x mTmG reporter mice. Upon tamoxifen treatment, the reporter switches from tdtomato (yellow) to GFP (cyan). GFP positive blood vessels do not show PLVAP expression (arrowheads). **c-e,** Violin plot depicting *Vegf* receptor levels in vWAT ECs in lean (CD) and obese (HFD) mice. **f,** Quantitative real-time PCR detection of *Vegf* receptors and **g,** *Vegfa* in vWAT ECs isolated from lean (n=6 mice) and obese (n=6 mice) animals. **h,** Representative confocal images of VEGFA (cyan) and isolectin B4 (magenta) staining in vWAT sections around large caliber vessels in lean (CD) or obese mice (HFD) and its **i,** quantification (CD n=6, HFD n=5 mice). **j,** Representative confocal images of VEGFA (cyan) and isolectin B4 (magenta) staining in vWAT sections in capillary areas in lean (CD) or obese mice (HFD) and its **k,** quantification (CD n=6, HFD n=6 mice). 4-5 high magnification fields per mice were quantified. Scale bars 100 µm. Data represents ± SEM, two-sided Mann-Whitney test (g), two-sided Welch's t-test (a, f, i, k). Source data are provided as a Source Data file.

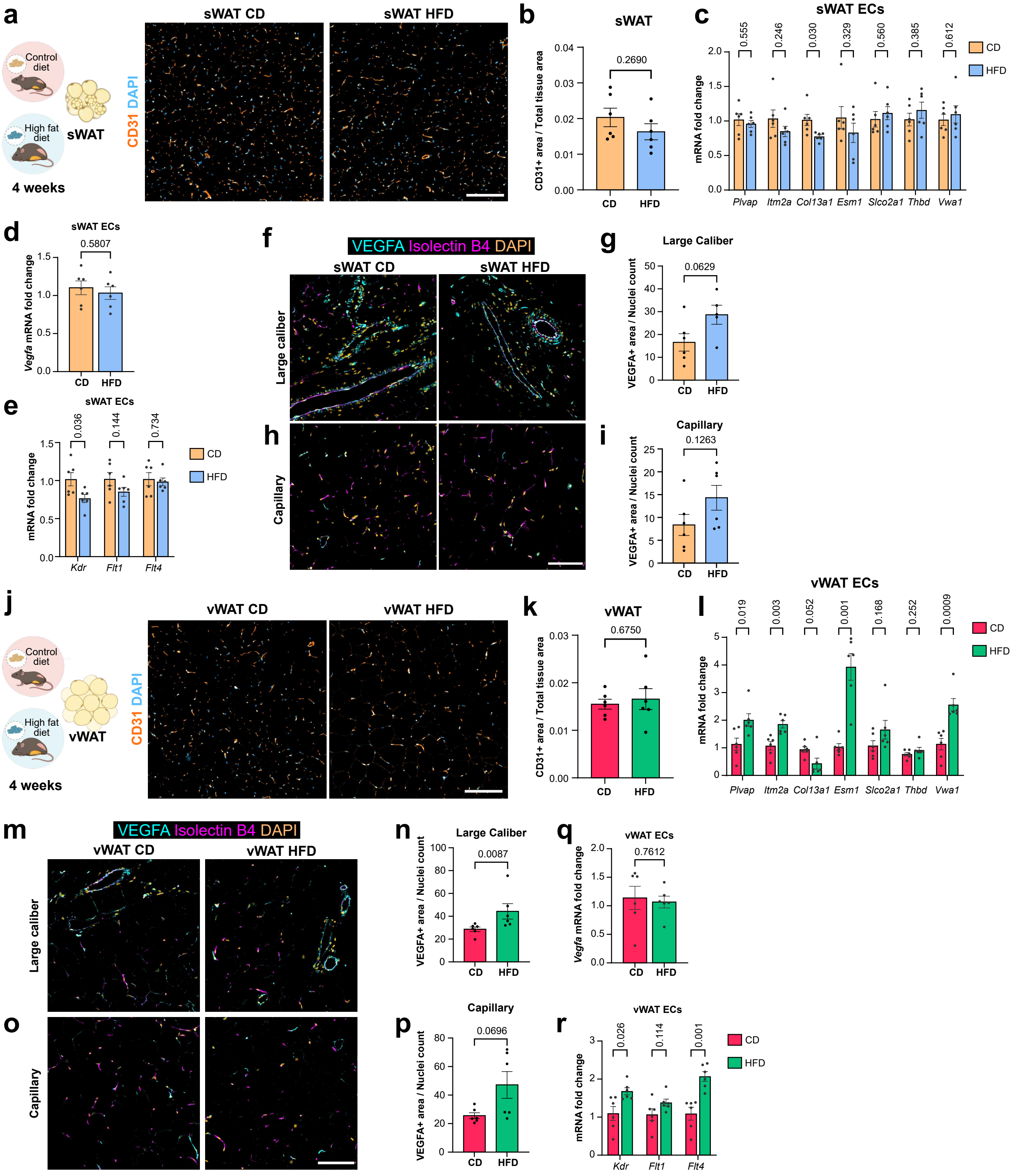

Supplementary Figure 8: Differential endothelial response during short-term high-fat diet.

a, Representative confocal images showing CD31+ (orange) blood vessels and DAPI+ (cyan) nuclei in tissue sections from subcutaneous white adipose tissue (sWAT) from mice fed either a control diet (CD) or high fat diet (HFD) for 4 weeks. **b,** Quantification of blood vessel (CD31+) area in sWAT (n=6 mice for CD and HFD). **c,** Quantitative real-time PCR detection of marker genes of fenestrations, **d,** *Vegfa* and **e,** *Vegf* receptors in sWAT endothelial cells (ECs) isolated from mice fed either CD or HFD (n=6 mice for each group). **f,** Representative confocal images of VEGFA (cyan) and isolectin B4 (magenta) staining in sWAT sections around large vessels in mice fed CD or HFD. **g,** Quantification of VEGFA+ area normalized to nuclei count around large vessels (CD n=6, HFD n=5 mice). **h,** Representative confocal images of VEGFA (cyan) and isolectin B4 (magenta) staining in sWAT sections from capillary area in mice fed CD or HFD. **i,** Quantification of VEGFA+ area normalized to nuclei count in capillary area (n=6 mice for each group). **j,** Representative confocal images showing CD31+ (orange) blood vessels and DAPI+ (cyan) nuclei in tissue sections from visceral WAT (vWAT) from mice fed either a CD or HFD for 4 weeks. **k,** Quantification of blood vessel (CD31+) area in vWAT (n=6 mice for each group). **l,** Quantitative real-time PCR detection of marker genes of fenestration in vWAT ECs isolated from mice fed either CD or HFD (n=6 mice for each group). **m,** Representative confocal images of VEGFA (cyan) and isolectin B4 (magenta) staining in vWAT sections around large vessels in mice fed CD or HFD. **n,** Quantification of VEGFA+ area normalized to nuclei count around large vessels (n=6 mice for each group). **o,** Representative confocal images of VEGFA (cyan) and isolectin B4 (magenta) staining in vWAT sections from capillary area in mice fed CD or HFD. **p,** Quantification of VEGFA+ area normalized to nuclei count in capillary area (n=6 mice for each group). **q,** Quantitative real-time PCR detection of *Vegfa* and **r,** *Vegf* receptors in vWAT ECs isolated from mice fed either CD or HFD (n=6 mice for each group). Scale bars 100 µm. Data represents ± SEM, two-sided Welch's t-test (b, c, d, e, g, i, k, l, p, q, r), two-sided Mann-Whitney test (n). Schematics created in BioRender. Hasan, S. (2025) https://BioRender.com/gsfwa7p. Source data are provided as a Source Data file.

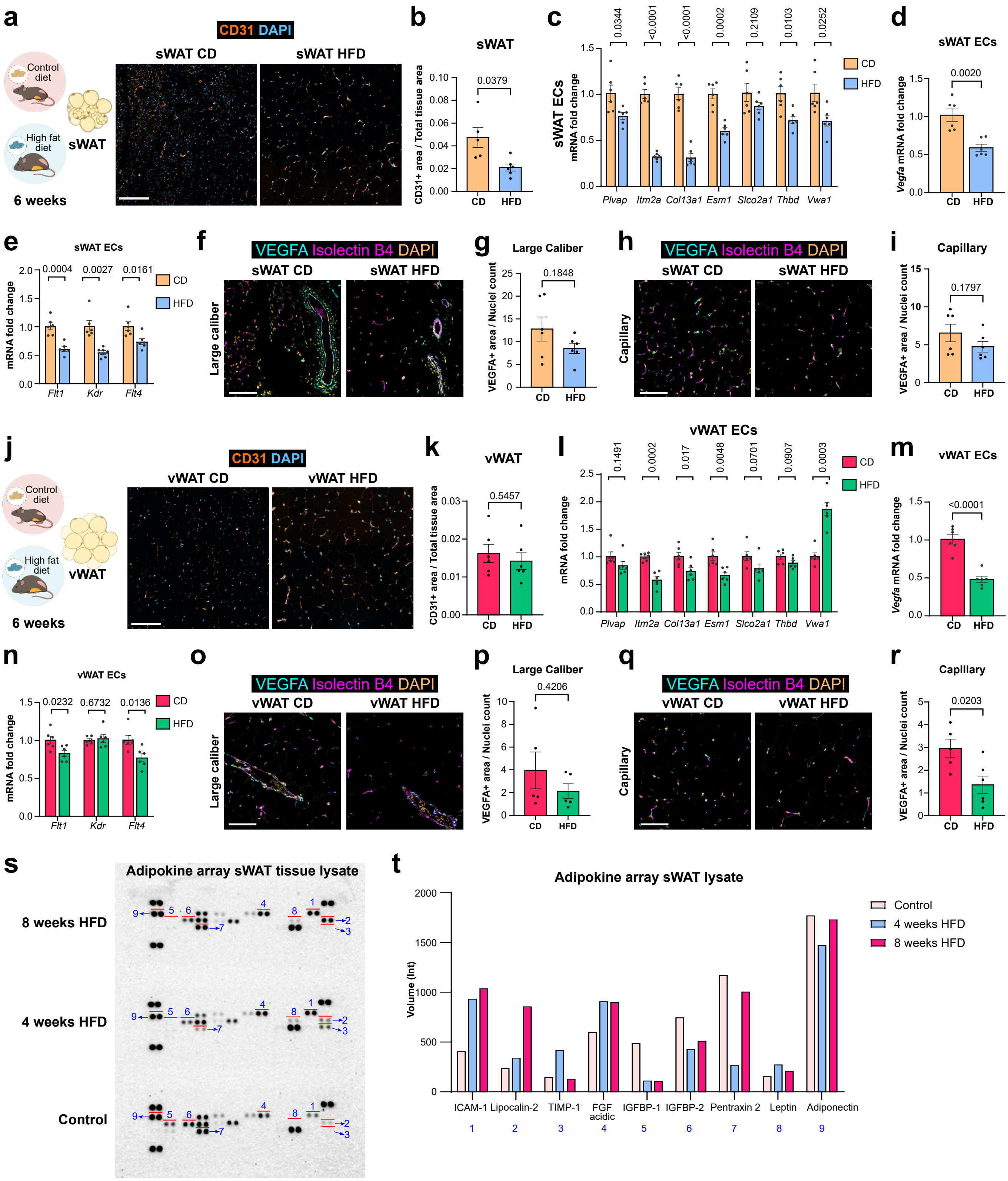

Supplementary Figure 9: Endothelial response during intermediate high-fat diet.

a, Representative confocal images showing CD31+ (orange) blood vessels and DAPI+ (cyan) nuclei in tissue sections from subcutaneous white adipose tissue (sWAT) from mice fed either a control diet (CD) or high fat diet (HFD) for 6 weeks. **b,** Quantification of blood vessel (CD31+) area in sWAT (CD n=5, HFD n=6 mice). Quantitative real-time PCR detection of **c,** marker genes of fenestrations, **d,** *Vegfa* and **e,** *Vegf* receptors in sWAT ECs isolated from mice fed either CD or HFD (n=6 mice for each group). **f,** Representative confocal images of VEGFA (cyan) and isolectin B4 (magenta) staining in sWAT sections around large vessels in mice fed CD or HFD. **g,** Quantification of VEGFA+ area normalized to nuclei count around large vessels (n=6 mice for each group). **h,** Representative confocal images of VEGFA (cyan) and isolectin B4 (magenta) staining in sWAT sections from capillary area in mice fed CD or HFD. **i,** Quantification of VEGFA+ area normalized to nuclei count in capillary area (n=6 mice for each group). **j,** Representative confocal images showing CD31+ (orange) blood vessels and DAPI+ (cyan) nuclei in tissue sections from visceral WAT (vWAT) from mice fed either a CD or HFD for 6 weeks. **k,** Quantification of blood vessel (CD31+) area in vWAT (n=6 mice for each group). Quantitative real-time PCR detection of **l,** marker genes of fenestration **m,** *Vegfa* and **n,** *Vegf* receptors in vWAT ECs isolated from mice fed either CD or HFD (n=6 mice for each group). **o,** Representative confocal images of VEGFA (cyan) and isolectin B4 (magenta) staining in vWAT sections around large vessels in mice fed CD or HFD. **p,** Quantification of VEGFA+ area normalized to nuclei count around large caliber vessels (n=5 mice for each group). **q,** Representative confocal images of VEGFA (cyan) and isolectin B4 (magenta) staining in vWAT sections from capillary area in mice fed CD or HFD. **r,** Quantification of VEGFA+ area normalized to nuclei count in capillary area (CD n=5, HFD n=6 mice). **s,** Adipokine array performed with sWAT tissue lysates from lean and obese mice at different time points. **t,** Quantification of adipokines in sWAT lysate (Tissue lysates from 5 mice were pooled for each group). Scale bars 100 µm. Data represents ± SEM, two-sided Welch's t-test (b, c, d, e, g, k, l, m, n, r), two-sided Mann-Whitney test (i, p). Schematics created in BioRender. Hasan, S. (2025) https://BioRender.com/gsfwa7p. Source data are provided as a Source Data file.

Supplementary Table 1: Information on Hashtag sequences

Sample name	Index - i7	
**SWAT_CD_a**	AGGAGATG	Subcutaneous white adipose tissue from lean mice (control diet)
**SWAT_CD_b**	GATGTGGT	
**SWAT_CD_c**	CTACATCC	
**SWAT_CD_d**	TCCTCCAA	
**SWAT_HFD_a**	CAATACCC	Subcutaneous white adipose tissue from obese (high fat diet) mice
**SWAT_HFD_b**	TGTCTATG	
**SWAT_HFD_c**	ACCACGAA	
**SWAT_HFD_d**	GTGGGTGT	
**EWAT_CD_a**	CTTTGCGG	Visceral white adipose tissue from lean mice
**EWAT_CD_b**	TGCACAAA	
**EWAT_CD_c**	AAGCAGTC	
**EWAT_CD_d**	GCAGTTCT	
**EWAT_HFD_a**	GCACAATG	Visceral white adipose tissue from obese mice
**EWAT_HFD_b**	CTTGGTAC	
**EWAT_HFD_c**	TGCACCGT	
**EWAT_HFD_d**	AAGTTGCA	
**SWAT_CD**	CCGCGGTT	Hashtag Library for each condition
**SWAT_HFD**	TTATAACC	
**EWAT_CD**	GGACTTGG	
**EWAT_HFD**	AAGTCCAA	

*EWAT = vWAT

10x Genomics SI-PCR primer (for 10x Genomics Single Cell 3P (v2 and v3) and 5P kits)
**5'AATGATACGGCGACCACCGAGATCTACACTCTTTCCCTACACGACGCTC**

Hashtag cDNA PCR additive primer	Used for amplifying Hashtag cDNA
**5'GTGACTGGAGTTCAGACGTGTGCTC**	

Illumina TruSeq D701_s primer (for HTO amplification; i7 index 1, shorter than the original D701 Illumina sequence)

	**Primer Sequence**	**Index Sequence**	
sWAT CD	CAAGCAGAAGACGGCATACGAGATAACCGCGGGTGACTGGAGTTCAGACGTGTGC	CCGCGGTT	Used fron preparing Hashtag library
sWAT HFD	CAAGCAGAAGACGGCATACGAGATGGTTATAAGTGACTGGAGTTCAGACGTGTGC	TTATAACC	
eWAT CD	CAAGCAGAAGACGGCATACGAGATCCAAGTCCGTGACTGGAGTTCAGACGTGTGC	GGACTTGG	
eWAT HFD	CAAGCAGAAGACGGCATACGAGATTTGGACTTGTGACTGGAGTTCAGACGTGTGC	AAGTCCAA	

Three mice were used per conditon and each one labelled with the following Hashtag antibody
TotalSeq™-A0301-A0303 anti-mouse Hashtag 1-3 Antibody from Biolegend

Hashtag (Biolegend Cat#)	**PCR handle**	**Barcode**	**Capture sequence**
1 (155801)	GTGACTGGAGTTCAGACGTGTGCTCTTCCGATCT	ACCCACCAGTAAGAC	BAAAAAAAAAAAAAAAAAAAAAAAAAAAAAA*A*A
2 (155803)	GTGACTGGAGTTCAGACGTGTGCTCTTCCGATCT	GGTCGAGAGCATTCA	BAAAAAAAAAAAAAAAAAAAAAAAAAAAAAA*A*A
3 (155805)	GTGACTGGAGTTCAGACGTGTGCTCTTCCGATCT	CTTGCCGCATGTCAT	BAAAAAAAAAAAAAAAAAAAAAAAAAAAAAA*A*A

Supplementary Table 2: Number of cells obtained from each mice after quality control, hash assignment and doublet removal

Sample	Number of Cells
EWAT_CD n1	687
EWAT_CD n2	784
EWAT_CD n3	1059
EWAT_HFD n1	1711
EWAT_HFD n2	1738
EWAT_HFD n3	2497
SWAT_CD n1	1328
SWAT_CD n2	1003
SWAT_CD n3	945
SWAT_HFD n1	1178
SWAT_HFD n2	1067
SWAT_HFD n3	815
**Total**	**14812**

*Data downsampled to 2530 cells

*EWAT = vWAT

Supplementary Table 3: Number of cells in each cluster per mice

	Cap I	Cap II	Venous	Arterial	Fenestrated	Angiogenic	Cap III
vWAT_CD n1	323	103	43	53	0	28	4
vWAT_CD n2	244	95	76	89	0	84	6
vWAT_CD n3	502	151	54	93	1	40	6
vWAT_HFD n1	327	457	99	211	0	100	18
vWAT_HFD n2	213	451	175	178	0	218	22
vWAT_HFD n3	468	839	267	244	0	187	25
SWAT_CD n1	66	67	222	190	204	40	16
SWAT_CD n2	29	18	177	149	288	23	9
SWAT_CD n3	73	46	163	147	173	17	8
SWAT_HFD n1	162	59	106	113	36	82	168
SWAT_HFD n2	39	16	209	66	77	33	297
SWAT_HFD n3	73	19	125	72	32	48	151

Number of cells in each cluster per mice after downsampling

	Cap I	Cap II	Venous	Arterial	Fenestrated	Angiogenic	Cap III
vWAT_CD n1	317	112	37	62	0	21	4
vWAT_CD n2	223	120	73	96	0	76	4
vWAT_CD n3	479	172	51	103	1	34	2
vWAT_HFD n1	89	228	41	104	0	49	5
vWAT_HFD n2	72	213	65	77	0	105	2
vWAT_HFD n3	123	382	116	113	0	91	4
SWAT_CD n1	16	82	166	148	147	34	10
SWAT_CD n2	7	37	131	108	228	18	8
SWAT_CD n3	24	83	123	106	131	11	1
SWAT_HFD n1	126	64	78	106	29	67	141
SWAT_HFD n2	25	24	168	55	58	29	256
SWAT_HFD n3	61	29	101	51	27	40	118

Supplementary Table 4: Top 50 Cap I marker genes

Wilcoxon rank-sum test with Benjamini–Hochberg P value correction

Gene	p_value	p_value_adjusted	avg_log2FC	pct.1	pct.2
Car4	0	0	1.592667707	0.933	0.546
Gpihbp1	0	0	1.053932506	0.995	0.905
Fabp4	0	0	0.999376018	1	0.998
Rgcc	6.44E-220	2.95E-215	1.009154699	0.979	0.809
Tcf15	6.01E-211	2.75E-206	1.119306156	0.94	0.742
Timp4	5.35E-205	2.45E-200	1.528890269	0.811	0.502
Cfd	8.53E-188	3.91E-183	3.08552365	0.456	0.144
Ubb	3.37E-174	1.54E-169	0.550274767	0.999	0.997
Car3	8.96E-157	4.10E-152	2.457416013	0.56	0.264
Eif1	1.15E-141	5.27E-137	0.488164733	0.997	0.996
Ppp1r2	6.94E-137	3.18E-132	0.760881583	0.957	0.908
Ctsl	1.38E-134	6.33E-130	0.766173758	0.96	0.925
Lims2	4.33E-115	1.98E-110	0.899372722	0.835	0.729
38231	1.64E-113	7.51E-109	0.945479652	0.828	0.672
Sult1a1	1.57E-112	7.21E-108	0.939858061	0.847	0.737
Hspb1	1.65E-102	7.57E-98	0.571881001	0.987	0.981
Kank3	7.66E-102	3.51E-97	0.845910331	0.83	0.74
Gng11	1.66E-97	7.61E-93	0.562543268	0.972	0.969
Aqp7	3.65E-93	1.67E-88	0.837431907	0.802	0.605
Ybx1	2.11E-92	9.66E-88	0.438014253	0.994	0.998
Sepp1	2.28E-91	1.04E-86	0.609413662	0.945	0.925
Lpar6	1.93E-84	8.85E-80	0.95362966	0.748	0.623
Sh3glb1	2.17E-84	9.93E-80	0.478606966	0.974	0.97
Cxcl9	3.34E-84	1.53E-79	1.127816568	0.575	0.349
AW112010	7.62E-83	3.49E-78	0.736110182	0.889	0.841
Chchd10	5.95E-81	2.73E-76	1.433768354	0.437	0.225
Dbi	2.26E-79	1.04E-74	0.944257361	0.814	0.794
Cd300lg	3.22E-76	1.48E-71	0.503045117	0.956	0.883
Cav2	4.20E-76	1.92E-71	0.624731295	0.887	0.87
Ctnnbip1	1.06E-75	4.85E-71	0.74471646	0.806	0.729
Tspan13	1.22E-75	5.57E-71	0.529260026	0.951	0.927
Jund	6.41E-75	2.94E-70	0.695100085	0.97	0.97
Fth1	1.33E-72	6.09E-68	0.285991733	0.999	0.999
Hrct1	2.21E-70	1.01E-65	0.886034503	0.703	0.561
Cav1	2.73E-70	1.25E-65	0.440569882	0.981	0.971
Mgll	3.01E-70	1.38E-65	0.402670076	0.986	0.971
Ly6c1	3.15E-69	1.44E-64	0.357514279	0.997	0.991
Cd81	4.67E-69	2.14E-64	0.406144497	0.986	0.987
Fscn1	1.97E-62	9.01E-58	0.47633346	0.659	0.479
Atp5j	3.48E-62	1.59E-57	0.42622582	0.958	0.965
Gpx4	3.18E-57	1.46E-52	0.456668792	0.939	0.943
Thrsp	1.80E-55	8.26E-51	0.947342932	0.65	0.535
C1qtnf9	4.59E-55	2.10E-50	0.630679139	0.824	0.761
Lpl	1.60E-51	7.32E-47	0.632891514	0.722	0.564
Dhrs3	1.54E-49	7.03E-45	0.770161322	0.652	0.573
Apoe	1.67E-48	7.63E-44	0.416556381	0.951	0.865
Sdpr	1.85E-48	8.47E-44	0.355876796	0.987	0.978
Cd36	1.06E-47	4.87E-43	0.279034094	0.997	0.98

Supplementary Table 5: Top-50 Cap II marker genes

Wilcoxon rank-sum test with Benjamini–Hochberg P value correction

Gene	p_value	p_value_adjusted	avg_log2FC	pct.1	pct.2
Cd36	0	0	0.906301854	1	0.979
Cdh5	8.52E-215	3.90E-210	0.718167237	0.994	0.959
Car4	4.46E-207	2.04E-202	0.986135022	0.938	0.546
Mgll	1.06E-205	4.85E-201	0.734301319	0.997	0.968
Cd300lg	9.43E-196	4.32E-191	0.78019174	0.99	0.874
Sptbn1	1.38E-184	6.34E-180	0.661284361	0.996	0.989
Lpl	3.53E-183	1.62E-178	1.162052506	0.872	0.523
Txnip	2.16E-178	9.90E-174	0.843703425	0.99	0.922
Rgcc	3.74E-156	1.71E-151	0.757150882	0.992	0.806
Aqp7	2.21E-155	1.01E-150	0.915233608	0.885	0.583
Cldn5	1.09E-147	4.99E-143	0.780915213	0.985	0.79
Sdpr	8.51E-145	3.90E-140	0.586491681	0.999	0.974
Tcf4	7.55E-140	3.46E-135	0.68131202	0.983	0.914
Btnl9	2.94E-139	1.35E-134	1.017959515	0.872	0.61
Tcf15	3.34E-137	1.53E-132	0.738279997	0.953	0.738
Cxcl9	1.13E-135	5.20E-131	1.232700695	0.672	0.323
Adgrf5	2.26E-131	1.04E-126	0.675270578	0.977	0.893
Gpihbp1	5.00E-131	2.29E-126	0.564977638	1	0.904
Plin2	1.72E-126	7.90E-122	0.891636052	0.842	0.59
Tjp1	6.00E-120	2.75E-115	0.818496636	0.89	0.715
Ablim1	1.86E-117	8.50E-113	0.784741269	0.904	0.727
Efnb1	2.43E-116	1.11E-111	0.988765752	0.768	0.499
Jup	1.32E-112	6.04E-108	0.901445554	0.784	0.509
Kdr	2.91E-108	1.33E-103	0.636634796	0.959	0.823
Angptl4	2.42E-104	1.11E-99	1.000686666	0.799	0.58
Etl4	1.25E-102	5.72E-98	1.101590592	0.665	0.398
Mllt4	2.27E-100	1.04E-95	0.70637632	0.908	0.769
Fabp5	8.62E-99	3.95E-94	0.891146682	0.955	0.872
Ssh2	1.10E-98	5.02E-94	1.348535371	0.489	0.226
Lpar6	1.20E-97	5.48E-93	0.788998361	0.839	0.598
Mylip	8.46E-96	3.87E-91	0.876912297	0.772	0.544
Ppp1r2	2.92E-93	1.34E-88	0.489643004	0.97	0.904
Timp4	3.55E-89	1.62E-84	0.717636603	0.796	0.507
Malat1	2.85E-88	1.31E-83	0.621515212	0.996	0.98
S1pr1	6.38E-88	2.92E-83	0.640558514	0.913	0.78
Tns1	1.35E-87	6.20E-83	0.792220227	0.803	0.603
Tspan13	3.54E-87	1.62E-82	0.515156825	0.977	0.92
Tmem245	1.53E-85	7.02E-81	1.119936519	0.596	0.354
Ablim3	2.06E-85	9.42E-81	0.84397393	0.71	0.448
Tmcc3	3.18E-84	1.46E-79	0.831323163	0.776	0.579
Lims2	1.90E-83	8.70E-79	0.612300392	0.875	0.719
Ctla2a	1.44E-81	6.60E-77	0.64848277	0.961	0.868
Xdh	1.51E-81	6.91E-77	1.058369155	0.574	0.32
Cav1	1.71E-81	7.83E-77	0.400674849	0.997	0.967
Rsad2	3.53E-79	1.62E-74	0.904323238	0.68	0.432
38231	1.94E-78	8.89E-74	0.567385912	0.864	0.662
Sh3bp5	5.97E-78	2.73E-73	0.602469373	0.878	0.721
Abcb1b	2.79E-76	1.28E-71	0.990881905	0.52	0.272

Supplementary Table 6: Top 50 Venous marker genes

Wilcoxon rank-sum test with Benjamini–Hochberg P value correction

Gene	p_value	p_value_adjusted	avg_log2FC	pct.1	pct.2
Ifitm3	0	0	1.008625225	1	0.995
Rpl23	0	0	1.002899989	1	0.993
Ifitm2	0	0	1.200163985	0.998	0.977
Aqp1	0	0	2.118408097	0.978	0.497
Ehd4	0	0	2.247458936	0.958	0.403
Plvap	0	0	1.584976005	0.957	0.44
Lrg1	0	0	3.441983071	0.934	0.237
Il6st	0	0	2.171738121	0.915	0.517
Chp2	0	0	2.352832699	0.861	0.262
Tmem252	0	0	2.905693384	0.841	0.261
Spint2	0	0	2.488999589	0.808	0.229
Csrp2	0	0	2.737716745	0.783	0.244
Prss23	0	0	2.784843622	0.779	0.192
Selp	0	0	5.038585395	0.731	0.047
Vwf	0	0	3.769754367	0.719	0.127
Bgn	0	0	2.406224671	0.716	0.189
Ptgs1	0	0	3.318836052	0.701	0.106
Rasa4	0	0	3.337798578	0.665	0.109
Vcam1	0	0	3.537966953	0.657	0.085
Cysltr1	0	0	4.736016184	0.623	0.037
Ackr1	0	0	5.763783175	0.553	0.034
Lgals3	0	0	3.398114888	0.546	0.076
Igf1	0	0	3.288829659	0.542	0.087
Ctnnal1	0	0	3.676874336	0.52	0.053
Pdia5	0	0	3.741445362	0.501	0.054
Lbp	0	0	4.492756092	0.421	0.029
Sele	0	0	4.820936325	0.415	0.029
Ret	0	0	5.169332685	0.311	0.009
Timp2	2.16E-307	9.90E-303	2.53428989	0.645	0.156
Rps3	1.07E-305	4.91E-301	1.139996412	0.999	0.979
Thsd7a	1.64E-302	7.49E-298	2.788563336	0.526	0.089
Rplp0	6.47E-301	2.96E-296	1.159684231	0.998	0.947
Fgl2	6.80E-301	3.12E-296	3.728921943	0.317	0.019
Fmo2	1.97E-297	9.04E-293	2.223643057	0.848	0.373
Rps11	1.73E-293	7.90E-289	0.845172072	0.998	0.996
Rps16	3.26E-289	1.49E-284	0.883210802	0.999	0.996
Rps24	3.12E-282	1.43E-277	0.820847751	1	0.998
Fbln2	1.02E-281	4.66E-277	1.745286569	0.835	0.303
Acer3	2.85E-281	1.31E-276	3.010338518	0.468	0.075
Rps9	1.96E-276	8.96E-272	0.967223493	0.999	0.978
Pgm5	4.39E-274	2.01E-269	3.647360024	0.328	0.027
Rps5	6.89E-274	3.15E-269	0.907184192	0.999	0.982
Mras	1.97E-269	9.03E-265	2.788326937	0.479	0.083
Rps20	2.11E-268	9.66E-264	0.844585998	0.999	0.997
Eln	4.03E-265	1.84E-260	2.172556791	0.666	0.193
Rpl10	1.33E-264	6.10E-260	1.126954749	0.997	0.942
2200002D0	5.76E-263	2.64E-258	2.256620719	0.653	0.187

Supplementary Table 7: Top 50 Arterial marker genes

Wilcoxon rank-sum test with Benjamini–Hochberg P value correction

Gene	p_value	p_value_adjusted	avg_log2FC	pct.1	pct.2
Tm4sf1	0	0	1.50202807	1	0.962
Fbln5	0	0	3.321439129	0.918	0.314
Sema3g	0	0	2.827824917	0.787	0.202
Hey1	0	0	3.304782721	0.708	0.13
Gja4	0	0	3.815450815	0.706	0.113
Col8a1	0	0	4.738780525	0.702	0.051
Stmn2	0	0	3.846109097	0.67	0.118
Vegfc	0	0	3.797423008	0.583	0.081
Fn1	0	0	3.226817346	0.454	0.06
Alox12	0	0	3.646176958	0.426	0.048
Alpl	0	0	5.185936413	0.366	0.017
St8sia6	0	0	4.334689959	0.36	0.023
Gja5	0	0	5.318219523	0.329	0.014
Car7	1.37E-292	6.28E-288	3.110369748	0.45	0.062
Bmx	1.40E-287	6.40E-283	4.473163906	0.305	0.019
Rgs10	2.41E-265	1.10E-260	3.941860749	0.309	0.024
Edn1	2.33E-246	1.07E-241	2.372814766	0.63	0.186
Ptprr	3.74E-246	1.71E-241	2.717613237	0.524	0.123
Eps8l2	2.00E-243	9.17E-239	2.76638881	0.463	0.089
Glul	1.31E-239	5.98E-235	2.057762856	0.81	0.41
Efnb2	1.43E-223	6.53E-219	1.897832096	0.733	0.294
Atp2a3	1.46E-208	6.68E-204	2.939365555	0.365	0.059
Ltbp4	1.17E-202	5.35E-198	1.425223832	0.909	0.611
Sdcbp2	6.03E-202	2.76E-197	3.21319895	0.329	0.048
Crispld1	5.23E-199	2.40E-194	2.460774209	0.453	0.105
Dusp26	1.40E-197	6.43E-193	2.339462034	0.49	0.127
Msx1	1.20E-194	5.49E-190	2.462832216	0.468	0.116
Mecom	7.71E-192	3.53E-187	1.881005364	0.718	0.333
Fbln2	9.34E-155	4.28E-150	1.549801274	0.714	0.327
Rbp7	2.47E-154	1.13E-149	1.480625527	0.884	0.697
Igfbp3	1.30E-152	5.96E-148	2.130403022	0.436	0.123
Pdcd4	1.71E-148	7.83E-144	1.766266898	0.679	0.336
Fads3	1.05E-145	4.80E-141	2.061113748	0.458	0.141
Epas1	6.58E-144	3.02E-139	0.909694601	0.957	0.848
Plat	3.93E-143	1.80E-138	2.386807932	0.4	0.112
Id1	5.36E-140	2.45E-135	0.929311016	0.967	0.836
Palld	5.99E-136	2.74E-131	2.276473988	0.389	0.108
Lrrc3b	1.61E-135	7.37E-131	2.480588852	0.309	0.067
Icam2	8.96E-131	4.10E-126	0.906803925	0.94	0.788
Slc9a3r2	1.49E-129	6.82E-125	0.86506699	0.958	0.844
Nudt4	1.05E-124	4.81E-120	1.846697306	0.762	0.519
Tsc22d1	7.38E-124	3.38E-119	0.874498601	0.953	0.794
Fhl1	2.99E-123	1.37E-118	1.641840096	0.52	0.199
Crip1	6.25E-123	2.86E-118	0.760057235	0.993	0.921
40787	3.68E-122	1.69E-117	1.607355775	0.611	0.296
Slc6a6	1.05E-121	4.79E-117	1.647104698	0.701	0.396
Fam181b	4.08E-121	1.87E-116	1.513107907	0.589	0.257

Supplementary Table 8: Top 50 Fenestrated marker genes

Wilcoxon rank-sum test with Benjamini–Hochberg P value correction

Gene	p_value	p_value_adjusted	avg_log2FC	pct.1	pct.2
Igfbp7	0	0	2.395026074	1	0.974
Plvap	0	0	3.166826539	0.995	0.478
Itm2a	0	0	5.855964913	0.878	0.063
Col13a1	0	0	6.494019124	0.874	0.025
Vwa1	0	0	3.303468721	0.865	0.217
Fam167b	0	0	3.309499103	0.853	0.179
Efhd1	0	0	3.546053486	0.741	0.09
Slco2a1	0	0	2.806385994	0.728	0.116
Pcp4l1	0	0	4.131557334	0.668	0.046
Ramp3	0	0	6.585990944	0.617	0.007
Esm1	0	0	4.967964088	0.614	0.04
Exoc3l2	0	0	3.643133973	0.599	0.056
RP24-333I1	0	0	7.45631205	0.556	0.006
Gas7	0	0	3.866389379	0.539	0.049
Slc16a1	0	0	3.950558585	0.485	0.033
Tmem35	0	0	6.577955693	0.414	0.006
St3gal5	0	0	3.983248481	0.411	0.028
Piezo2	0	0	4.506156958	0.385	0.018
Penk	0	0	6.835092021	0.362	0.012
Ces2e	0	0	6.019800795	0.359	0.006
Pacsin1	0	0	7.704766692	0.332	0.002
Fam117a	2.94E-306	1.35E-301	3.525807973	0.456	0.039
Tnfaip2	1.03E-305	4.70E-301	3.316404625	0.639	0.101
Nr5a2	1.53E-288	7.02E-284	3.260326688	0.612	0.096
Cxcl12	1.40E-281	6.42E-277	2.496323397	1	0.886
Ppap2b	1.27E-277	5.82E-273	2.301427957	0.998	0.884
Col15a1	5.09E-271	2.33E-266	2.422444919	0.85	0.236
Ehd4	8.47E-265	3.88E-260	2.343042632	0.969	0.447
Fxyd5	3.60E-254	1.65E-249	2.045187857	0.966	0.482
Mef2c	4.27E-251	1.96E-246	2.389094482	0.971	0.675
Tbxa2r	4.51E-246	2.07E-241	3.571928796	0.354	0.028
Cd24a	8.98E-243	4.11E-238	2.520391927	0.543	0.08
Gm13889	4.46E-241	2.04E-236	2.566930198	0.655	0.132
Thbd	6.88E-241	3.15E-236	2.346558887	0.91	0.352
Mlf1	2.55E-240	1.17E-235	3.487703491	0.348	0.028
Lamb1	1.42E-234	6.52E-230	2.378376774	0.633	0.118
Fut8	1.20E-227	5.50E-223	2.655897306	0.543	0.088
Slc27a3	8.38E-225	3.84E-220	2.480006802	0.631	0.127
Ace	6.19E-223	2.84E-218	2.282429148	0.779	0.222
Tmem176a	1.33E-221	6.09E-217	2.272390506	0.936	0.478
Serpinb1a	1.15E-217	5.27E-213	2.37814838	0.617	0.124
Flt1	2.20E-213	1.01E-208	1.525370013	0.994	0.831
6430548M0	3.18E-213	1.45E-208	2.91417299	0.42	0.053
Sema7a	1.09E-205	4.97E-201	2.208602579	0.75	0.216
Smco4	1.66E-205	7.60E-201	1.694217304	0.974	0.68
Scn1b	9.49E-203	4.35E-198	2.101873449	0.723	0.19
Lrrc3b	3.87E-197	1.77E-192	2.444315799	0.465	0.071

Supplementary Table 9: Top 50 Angiogenic marker genes

Wilcoxon rank-sum test with Benjamini–Hochberg P value correction

Gene	p_value	p_value_adjusted	avg_log2FC	pct.1	pct.2
AA467197	0	0	4.107963588	0.718	0.067
Mest	0	0	3.944449403	0.692	0.088
Pgf	0	0	5.775214327	0.668	0.026
Apln	0	0	5.094828614	0.611	0.038
Nid2	0	0	4.381231359	0.58	0.044
Kit	0	0	3.807641319	0.569	0.043
Gng2	4.72E-301	2.16E-296	3.816175688	0.433	0.034
Tmsb10	1.31E-292	5.98E-288	2.749698463	0.998	0.837
Fscn1	4.73E-290	2.17E-285	2.770116662	0.981	0.478
Vim	3.19E-237	1.46E-232	2.18403068	1	0.911
Cd109	3.40E-237	1.56E-232	3.521233873	0.369	0.032
Dpysl3	1.72E-236	7.87E-232	3.049624178	0.588	0.105
Trp53i11	1.41E-229	6.48E-225	2.305949395	0.888	0.315
Col4a1	2.94E-226	1.34E-221	2.096815503	0.995	0.819
Prnp	2.90E-222	1.33E-217	2.369743134	0.946	0.512
Lgals1	8.58E-216	3.93E-211	2.428382283	0.92	0.418
F2r	2.42E-215	1.11E-210	2.709365084	0.51	0.077
Cd34	4.22E-209	1.93E-204	1.791042573	0.976	0.633
Cxcr4	6.53E-200	2.99E-195	2.944830892	0.41	0.051
Tnfaip8l1	2.56E-199	1.17E-194	2.653180858	0.6	0.128
Adm	6.07E-197	2.78E-192	2.630478379	0.493	0.078
Pfn1	4.29E-191	1.96E-186	1.443296861	0.995	0.932
Tbc1d16	8.53E-190	3.91E-185	3.288543217	0.301	0.026
Col4a2	1.32E-187	6.04E-183	1.882250327	0.964	0.686
Gnai2	1.44E-187	6.60E-183	1.053848395	1	0.977
Lxn	8.79E-186	4.03E-181	2.333671297	0.756	0.255
Serpinh1	2.31E-185	1.06E-180	1.914992763	0.943	0.652
Arl4c	2.07E-183	9.47E-179	2.918875964	0.37	0.045
Sparc	1.90E-181	8.71E-177	1.251363252	1	0.994
Gnb4	7.40E-176	3.39E-171	2.261237861	0.787	0.279
Actb	8.39E-176	3.84E-171	1.423032791	1	0.998
Fkbp1a	1.38E-175	6.31E-171	1.251176858	0.991	0.911
Mpp6	1.47E-170	6.72E-166	2.613695442	0.467	0.082
Rhoc	5.29E-169	2.42E-164	1.760594005	0.953	0.755
Cd82	3.16E-166	1.45E-161	2.945588474	0.351	0.045
Tubb6	2.73E-163	1.25E-158	2.953443169	0.408	0.066
Mmp14	8.00E-161	3.66E-156	2.567568252	0.439	0.077
Mtch1	9.93E-159	4.55E-154	1.444887902	0.96	0.71
Angpt2	3.55E-157	1.63E-152	2.511380195	0.448	0.081
N4bp3	1.64E-156	7.53E-152	1.813360488	0.799	0.283
Col28a1	3.06E-155	1.40E-150	2.325230249	0.526	0.114
Smad1	6.00E-155	2.75E-150	1.93519083	0.834	0.372
Tnc	6.42E-153	2.94E-148	2.719748677	0.31	0.037
Gpc4	3.68E-152	1.68E-147	2.897153408	0.303	0.036
Tpm4	1.69E-147	7.72E-143	1.411390268	0.955	0.72
Map1b	2.24E-145	1.02E-140	2.000827893	0.69	0.221
Rarres1	2.56E-144	1.17E-139	2.858824025	0.313	0.041

Supplementary Table 10: Top 50 Cap III marker genes

Wilcoxon rank-sum test with Benjamini–Hochberg P value correction

Gene	p_value	p_value_adjusted	avg_log2FC	pct.1	pct.2
Col1a1	0	0	7.95844026	0.619	0.01
Col1a2	0	0	7.003965236	0.64	0.02
Col3a1	0	0	6.756368987	0.786	0.039
Dcn	0	0	6.448128194	0.529	0.017
Meg3	0	0	6.350260707	0.392	0.005
Col6a1	0	0	5.914790361	0.531	0.02
Prrx1	0	0	5.795418052	0.331	0.01
Col6a3	0	0	5.760986858	0.374	0.013
Postn	0	0	5.522771916	0.66	0.034
Col6a2	0	0	5.259543955	0.41	0.019
Myh11	0	0	5.052211319	0.466	0.027
Igfbp5	0	0	5.024162844	0.559	0.03
Aspn	0	0	4.862333663	0.581	0.035
Acta2	0	0	4.75775992	0.712	0.05
Tpm2	0	0	4.57643191	0.464	0.034
Gucy1a3	0	0	4.417112994	0.532	0.041
Gsn	0	0	4.359409866	0.878	0.151
Myl9	0	0	3.846505809	0.655	0.077
Serping1	0	0	3.678623528	0.612	0.086
Rgs5	0	0	2.997505324	0.887	0.199
Cygb	1.26E-285	5.79E-281	3.710101421	0.559	0.077
Col5a3	5.11E-261	2.34E-256	4.99735293	0.309	0.016
Rarres2	5.34E-258	2.44E-253	3.584874062	0.525	0.073
Zeb2	3.85E-243	1.76E-238	3.662516682	0.469	0.059
Steap4	9.71E-238	4.45E-233	4.408909326	0.327	0.023
Gucy1b3	3.33E-231	1.53E-226	4.268423676	0.3	0.019
C1s1	2.31E-228	1.06E-223	4.54820916	0.317	0.023
Tagln	4.89E-225	2.24E-220	1.862077771	0.779	0.197
Col5a2	1.09E-218	4.97E-214	3.958789668	0.335	0.028
Ndufa4l2	3.25E-197	1.49E-192	3.160438002	0.466	0.074
Cald1	2.68E-192	1.23E-187	2.249637761	0.897	0.5
Serpine2	1.07E-190	4.92E-186	3.922404294	0.313	0.029
Mgp	2.88E-176	1.32E-171	0.828672321	0.513	0.097
Abcc9	2.72E-162	1.25E-157	3.932180793	0.302	0.034
Gm37376	5.83E-157	2.67E-152	3.057623376	0.46	0.094
Anxa1	5.07E-137	2.32E-132	2.337869702	0.638	0.227
Actb	3.18E-135	1.46E-130	0.727806648	1	0.998
Phlda1	1.93E-131	8.82E-127	2.896962742	0.415	0.09
Pcolce	4.27E-127	1.96E-122	3.022415128	0.428	0.102
Lgals1	6.06E-123	2.78E-118	1.637395261	0.833	0.427
Fn1	3.49E-118	1.60E-113	2.194680393	0.43	0.096
Rasl11a	2.15E-117	9.84E-113	2.171384406	0.477	0.127
Bgn	3.38E-116	1.55E-111	1.562568137	0.665	0.24
Gm13889	2.02E-111	9.24E-107	2.454443884	0.502	0.151
Cd63	8.13E-108	3.72E-103	1.700371538	0.719	0.33
Mt1	5.88E-102	2.69E-97	2.094783648	0.405	0.101
S100a6	1.68E-94	7.69E-90	0.989072322	0.984	0.774

Supplementary Table 11: List of primers used for qRT-PCR

Gene	Forward Primer	Reverse Primer
mCph	ATGGTCAACCCCACCGTG	TTCTGCTGTCTTTGGAACTTTGTC
mCol1a1	CGATGGATTCCCGTTCGAGT	GAGGCCTCGGTGGACATTAG
mCol1a2	TGGTGATAAAGGGCACAGGG	ACCATGTAGGCCAGCAAGAC
mCol3a1	GACCTAAGGGCGAAGATGGC	AAGCCACTAGGACCCCTTTCT
mPlvap	CGTCAAGGCCAAGTCGCT	AGGGTTGACTACAGGGAGCC
mItm2a	AAGATCGCCTTCAACACCCC	GGGACAACTCTGAGCTCCTTG
mCol13a1	AAGGGAGAAGCAGGCCTAGA	GGAGTACCAGGCAATCCCAG
mEsm1	CCTGGAGAAACCTGCTACCG	GGTGCCATAGGGACAGTCTTT
mSlco2a1	CATGATGGTCCTACGTGTGGT	TCATAATACGCACAGGCCCC
mThbd	AAGCCATGCGAGACTGAGAC	CGCGAGTATTCACCGTCAGA
mVwa1	GGGGCTCCATAACTGATGCG	CTGTTGGCGTCTTGTGGTTG
mKdr	GGCGGTGGTGACAGTATCTT	GTCACTGACAGAGGCGATGA
mFlt1	GGATGCAGGGGACTATACGA	CCATACACGGTGCAAGTGAG
mFlt4	CCCCTCCAACCCTGAACATC	GCTGTCCCCTGCAGGATATG
mVegfa	CTGGACCCTGGCTTTACTGC	TGAACTTGATCACTTCATGGGACT
